# Effect of hyper-parameters on the performance of ConvLSTM based deep neural network in crop classification

**DOI:** 10.1371/journal.pone.0275653

**Published:** 2023-02-09

**Authors:** Awab ur Rashid Durrani, Nasru Minallah, Najam Aziz, Jaroslav Frnda, Waleed Khan, Jan Nedoma

**Affiliations:** 1 Department of Computer Systems Engineering, University of Engineering and Technology Peshawar (UETP), Peshawar, Pakistan; 2 National Center for Big Data and Cloud Computing (NCBC), University of Engineering and Technology Peshawar (UETP), Peshawar, Pakistan; 3 Department of Quantitative Methods and Economic Informatics, Faculty of Operation and Economics of Transport and Communication, University of Zilina, Zilina, Slovakia; 4 Department of Telecommunications, Faculty of Electrical Engineering and Computer Science, VSB - Technical University, Ostrava-Poruba, Czechia of Ostrava, Ostrava-Poruba, Czechia; Newcastle University, UNITED KINGDOM

## Abstract

Deep learning based data driven methods with multi-sensors spectro-temporal data are widely used for pattern identification and land-cover classification in remote sensing domain. However, adjusting the right tuning for the deep learning models is extremely important as different parameter setting can alter the performance of the model. In our research work, we have evaluated the performance of Convolutional Long Short-Term Memory (ConvLSTM) and deep learning techniques, over various hyper-parameters setting for an imbalanced dataset and the one with highest performance is utilized for land-cover classification. The parameters that are considered for experimentation are; Batch size, Number of Layers in ConvLSTM model, and No of filters in each layer of the ConvLSTM are the parameters that will be considered for our experimentation. Experiments also have been conducted on LSTM model for comparison using the same hyper-parameters. It has been found that the two layered ConvLSTM model having 16-filters and a batch size of 128 outperforms other setting scenarios, with an overall validation accuracy of 97.71%. The accuracy achieved for the LSTM is 93.9% for training and 92.7% for testing.

## Introduction

Knowledge enables intelligence. Getting knowledge into computers has always been a challenge for computer scientists. Rule or heuristic based knowledge can easily be transferred into computers using conventional programming paradigms, however transferring the experience or expertise based knowledge is quite challenging. Therefore, the paradigm is shifting towards learning automatically through examples or machine learning. It has enabled machines (computers) to figure out the rules by itself, mimicking the human ability to learn [1].

Since the previous decade, a machine learning algorithm modelled on the human brain called artificial neural networks has been knocking over the benchmarks in various fields of computer science [2]. It is based on a layered network of artificial neurons [3], that can learn different representations of data and make decisions based upon them. It has automated the feature engineering phase of learning, hence making it a more scalable and powerful tool by eliminating the need for domain knowledge or human experts [4]. It has made into a whole sub-field of itself called deep learning, where ‘deep’ is referring to the fact that it involves Artificial Neural Network (ANN) containing two or more hidden layers [5]. The main factors involved in the recent revolution is availability of huge amounts of data due to the emergence of big internet companies that gather huge variety, velocity and veracity of data as well as higher computational power due to the availability of Graphics Processing Units (GPUs) and Tensor Processing Units (TPUs) [6]. These internet companies are also investing in research and development, hence resulting in easy to use powerful tools like Tensorflow [7], Keras [8] and Pytorch [9] for the development of deep learning models.

Since the advent of AlexNet [10] in 2012, deep learning has revolutionized the field of computer vision, natural language processing and speech recognition. It has knocked over all the benchmarks in the fields one by one. Recently, it is finding its way in other fields as well.

Remote sensing has its own challenges that deep learning based algorithms have to address [11–13]. Unlike computer vision where the camera sensor mostly provides only 3 spectral bands Red, Green and Blue, the remote sensing based satellites are equipped with either multi-spectral or hyper-spectral sensors [14]. These sensors can capture a wide range of spectral bands that are beyond visible range on the electromagnetic spectrum. Each spectral band has different spatial resolutions, therefore choice of interpolation of the bands to align them with each other can result in different results [15]. The remote sensing data also involves multi-modal data as the satellite imagery data is Geo-tagged. Each pixel of the imagery has latitude and longitude information attached with it. Utilizing the Geo-referenced data along with the spectral intensities is a challenge for deep learning based architectures [16]. Moreover, each satellite has different capabilities in terms of temporal resolution i.e., the ability of a satellite to image the whole earth in a certain amount of time. Temporal images are crucial for land cover classification and segmentation tasks, as it provides change in the land cover with time [17].

Despite having nonparallel applications of deep learning in computer vision, its use in Land Use Land Cover (LULC) analysis using remote sensing is still limited. One of the key drawbacks, researchers and machine learning enthusiasts are facing is the availability of localized crop data-sets. In developing countries like Pakistan, little to non-enhancements in the crop statistics generation has been seen in the last decades. Traditional methods comprising of field surveying and unrealistic approaches for vegetation fields acreage estimation are carried out seasonally. In-situ data collected in our area under observation is mostly unbalanced (Fig 5), thus resulting in a rise of over-fitting in certain categories.

Against this background, we have proposed the ConvLSTM and LSTM models. The models achieved accuracy of 97.71% and 92.7% respectively. Hence concluding the better performance of ConvLSTM over LSTM in our experimental setup, and has the following research contribution.

- Our work creates a synergy of PlanetScope and Sentinel-2 (having different Spatial and Spectral resolutions) to effectively explore their advantage, while performing our experiments on the acquired data, to achieve best performance gain.
- Another important aspect of our research work is model architecture of the proposed ConvLSTM. The ConvLSTM model performs convolution operation instead of element-wise multiplication as in the case of LSTM model. In our case, the data is spectro-temporal and we proposed a model based on ConvLSTM layers, and compared its performance with the LSTM model over various sets of hyperparameters.
- Our work also investigated the best setting of hyperparameters for the implemented deep learning models. As hyperparameters can be set in various settings, they also impact the performance of the model. Hence, through this study, we utilized different settings for various hyperparameters. The three hyperparameters that are chosen for experimentation includes;
- Batch Size
- Filter Size
- Number of Layers

### Related work

Convolutional neural networks are a class of ANNs that has proven their effectiveness in the area of image recognition and classification. Recently, deep CNNs [18] are making inroads in other areas and are performing well. Vrskova *et al*., [19] performed a hyper-parameter case study on in buildings vegetation detection using CNN. A low altitude drone collected database was created in Zurich (Switzerland). Vrskova *et al*. [19] describes hyper-parameters tuning as the biggest dependency on deep learning. A ConvLSTM is used for the detection for face mask in video stream, comparing Genetic algorithm. Grid search and Bayesian optimization. The primary contribution of the work is hyper-parameter tuning using filter size, number of filters, number of epochs, training optimization algorithm and batch size.

Alex *et al*. [10] implemented a deep CNN trained through the imagenet dataset. In their study, they used non-saturating neurons for faster training and efficient GPU implementation for the convolution operation. They achieved considerably better performance with their deep CNN in comparison to other state-of-the-art techniques.

To predict the future rainfall intensity in a region, Shi *et al*. [20], formulated precipitation nowcasting as a spatio-temporal sequence forecasting problem in which input and output are both spatio-temporal sequences. They proposed and implemented Convolutional Long Short-Term Memory (ConvLSTM) by extending the Fully Connected LSTM (FC-LSTM) [20] to have convolutional structure in input-to-state and state-to-state transitions. They have shown in their study that the proposed model was better in capturing spatio-temporal correlation and outperforms the state-of-the-art ROVER algorithm [20] as well FC-LSTM in precipitation nowcasting.

A FC-LSTM network based on fully connected neural net and convolutional LSTM is developed and implemented by Teimouri et al. [21] for recognizing various agricultural crops in Synthetic-Aperture Radar (SAR) imaging data.

Their proposed structure was able to identify different crops with high performance. However, the classification confidence in the border region of the field was relatively lower than in the center of the fields. To model long term dependencies in the spectral dimension of hyperspectral imaging or time dimension of videos, a special deep learning structure of LSTM has shown promising abilities. Hu *et al*. [22] proposed two novel methods for the extraction of more discriminative spatio-spectral features by exploiting the convolutional LSTM. To model long range dependencies, the 2-D extended architecture of LSTM is considered for building the Spatial-Spectral ConvLSTM 2-D Neural Network (SSCL2DNN). To better preserve the intrinsic structure information of the hyperspectral data, the Spatial-Spectral ConvLSTM 3-D Neural Network (SSCL3DNN) is proposed by extending LSTM to the 3-D version for further improving the classification. Their proposed models achieved better classification performance on HSI datasets in comparison to other standard methods. Generative Adversarial Networks (GAN) is a new framework for generative model estimation through an adversarial network which is proposed by Goodfellow *et al*. [23, 24]. They trained a generative model that captures distribution, and a discriminative model that estimates the probability of a sample that came from training data rather than a generative model. Their experiments demonstrated potential of the framework by quantitative and qualitative evaluation of generated samples.

A hybrid CNN is used in the work of Ghaderizadeh et al. They found that hybrid CNN reduces the model’s complexity and can be effective in the presence of noise. They utilized the Adam optimizer and find that it shortens training time and improves network optimization. Alireza et al. [25] utilized various machine learning algorithms for yield forecasting of the barley crop in southern Iran. It was found that the Gaussian process regression algorithm that they have implemented outperformed four other machine learning techniques in their research settings [26]. Ghaderizadeh et al. proposed MDBRSSN (Multiscale dual branch residual spectral spatial network) model and compared their performance with other implemented models which are 2D-CNN and GAP-2D. The focus of their study was on HSI (hyperspectral imaging) classification. They have performed their experiments on four different datasets, and find out that their proposed model outperformed the compared state of the art methods, especially, in the case of limited training samples [27]. Sharifi et al. estimated the maize nitrogen uptake. They have utilized sentinel-2 data and compared the performance over various bands for nitrogen uptake at peak greenness. Their study suggests that near infrared and red-edge bands in vegetation indices would be the better predictor for maize nitrogen levels [28].

## Materials and methods

**Fig 1:**
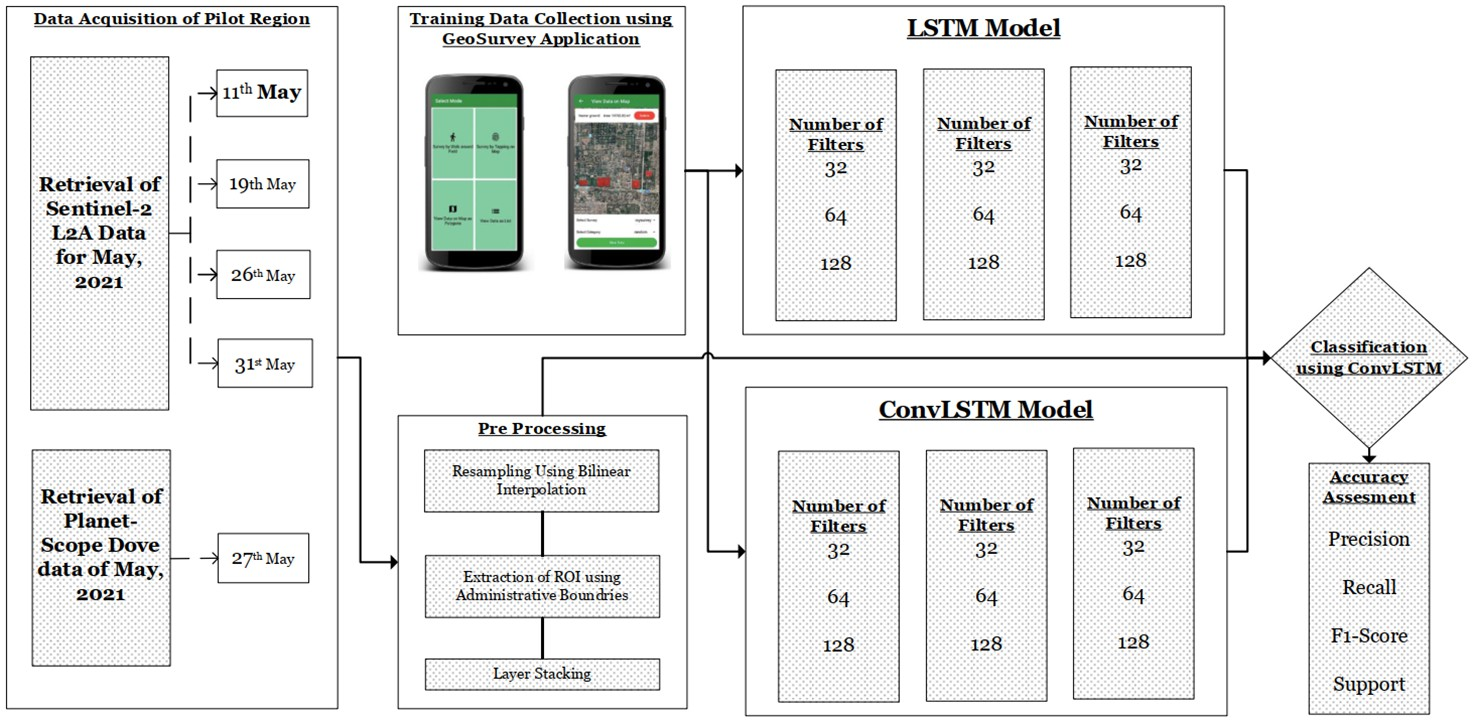
Flow chart of the methodology used in the study.

Fig 1 provides and overview of the methodology used in the study. Training data is collected using our developed survey application ‘Geo Survey’. For satellite data, a synergy of Planet-Scope due to its Very High Resolution (VHR) and Sentinel-2 has been created for classification.

### Pilot region

**Fig 2:**
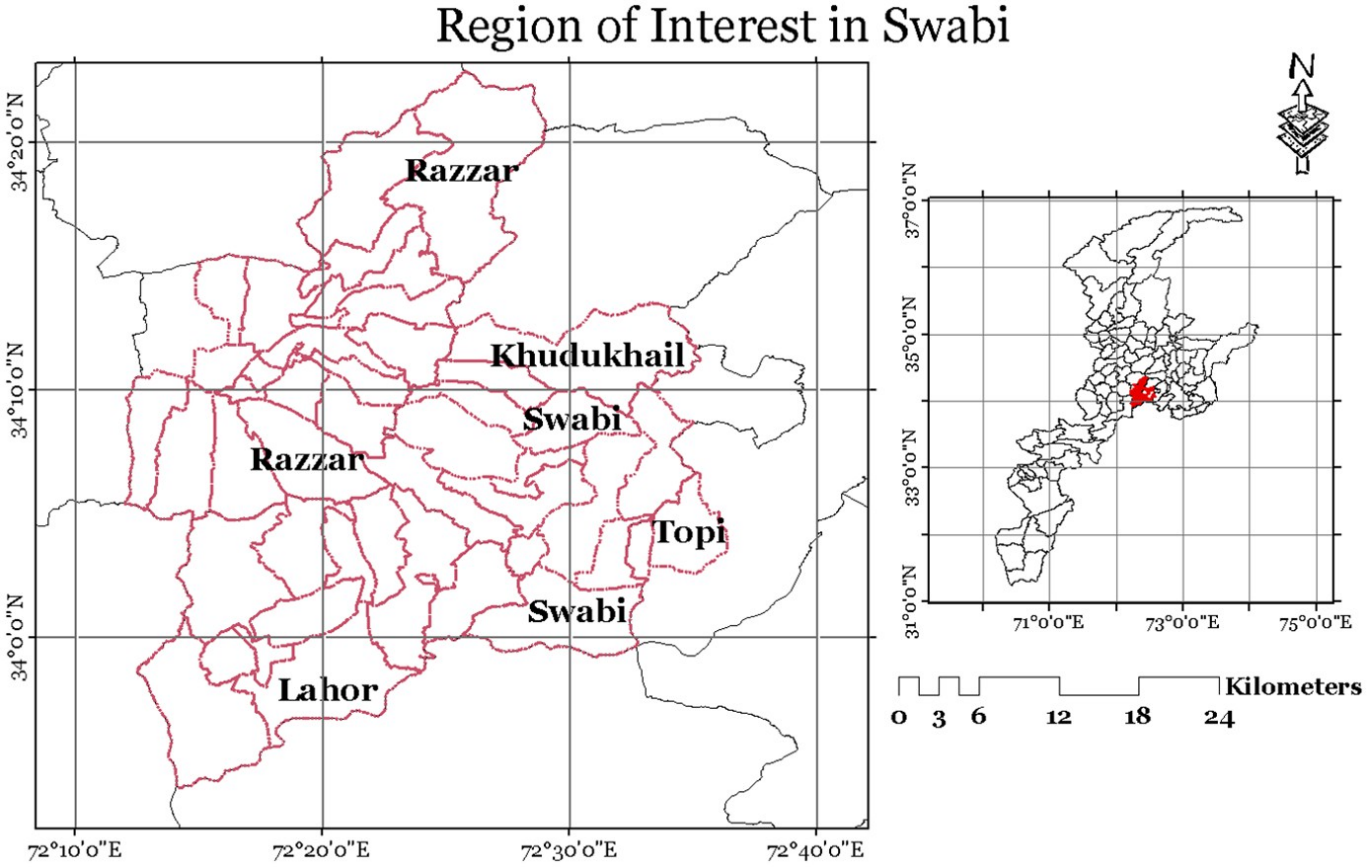
Locality map (generated using administrative boundaries and no copyright permission is required) of regions of interest. https://www.openstreetmap.org/map=5/30.671/69.360.

The pilot region for this study is located in the Khyber Pakhtunkhwa (KP) province of Pakistan. More specifically, for our experimentation work in KP province, we selected specific areas of District Swabi as presented in Fig 2. This area has wide arable land and a diverse vegetation environment. The region is known for high quality *tobacco* crop, *sugarcane*, *wheat* and various other crops which are considered as a great revenue generation potential for the KP province in-terms of taxable income. The locality map is shown in Fig 2.

### Remote sensing multi-spectral data

We used the Sentinel-2 and Planet-Scope satellites remotely sensed data in our experimental setup.

#### Sentinel-2

This is an open data satellite imagery, obtained from the satellite Copernicus open hub Sentinel-2 [29]. Sentinel-2 is an earth observation mission from the Copernicus Program that systematically acquires optical imagery at a high spatial resolution (10 m to 60 m) over land and coastal *water*s. In our experimental study focusing on the classification of *tobacco* crops, we considered our pilot region’s remotely sensed imagery, acquired on 5, 11, 26 and 31 May 2021, while keeping in mind the phonological period of *tobacco* crops.

#### Planet-Scope

With over 200 satellites currently in orbit, the Planet-Scope constellation makes up the largest commercial satellite fleet in history, capturing regular images of the Earth’s entire landmass [30, 31]. The sensors are capable of capturing four different multi-spectral bands with a resolution of 3-5 metres, including red, green, blue, and near-infrared multi-spectral bands, which are reasonable for analysing and monitoring changes in vegetation and Trees cover. Planet-Scope is a commercial satellite and its data can be bought from Planet Inc. Planet-Scope [32] imagery of the pilot area (acquired on 27 May 2021). Fig 4 displays timeline of the photos of regions of interest that were obtained.

### Ground survey for data collection

**Fig 3:**
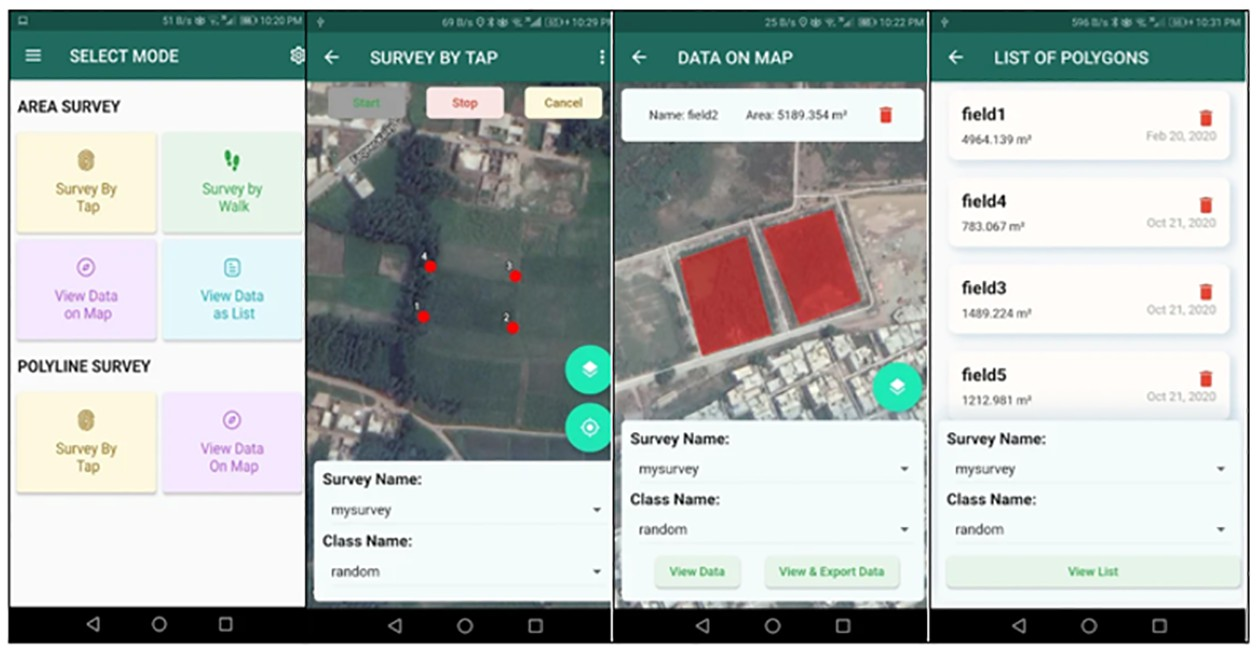
GeoSurvey application with different modes of polygon selection.

Pilot area ground data collection surveys were performed using the locally developed GeoSurvey application [33]. In Fig 3, a brief description of the GeoSurvey application is pictorially provided showing different modes of polygon selection using the application. The developed GeoSurvey framework is native and uses the programming language of JAVA. The data from the survey is stored in the real-time Google Firebase database. Firebase data is downloaded in the format of JavaScript Object Notation (JSON) and translated to Keyhole Markup Language (KML) using indigenous python scripting. The database which is used for data storage is MySQL. Finally, for training and evaluating the efficiency of the proposed model, KML is translated into shapefiles using ARCGIS. Compared to other conventional approaches, our survey application proved to be cost-effective and time-efficient due to the option of retrieving a polygon by encircling or interactively selecting various points. The underlying ground cover was divided into five separate groups in our experimental work, including *Urban*, *Wheat*, *Tobacco*, *Water* and *Other Vegetables*.

**Fig 4:**
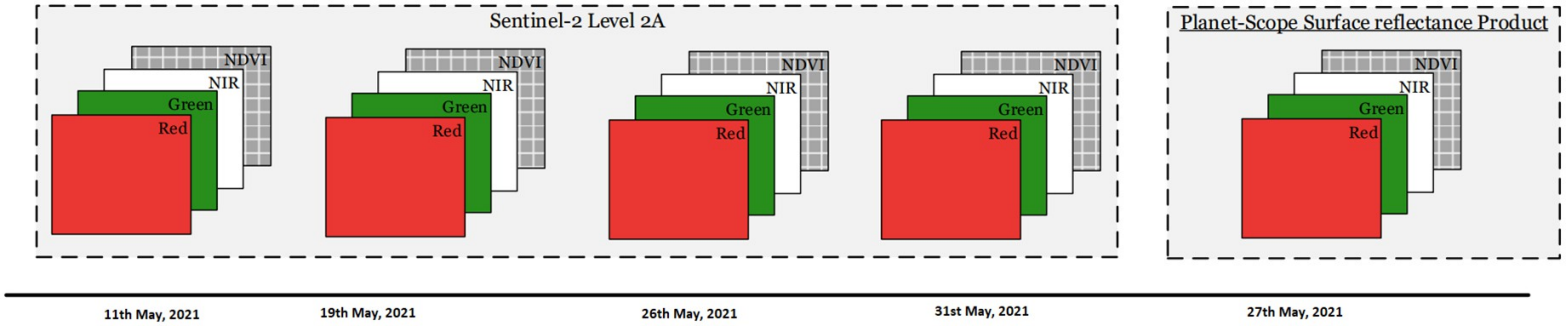
Timeline of the acquired images of the regions of interest.

The timeline of the acquired images of the pilot region is depicted in Fig 4.

### Dataset

**Fig 5:**
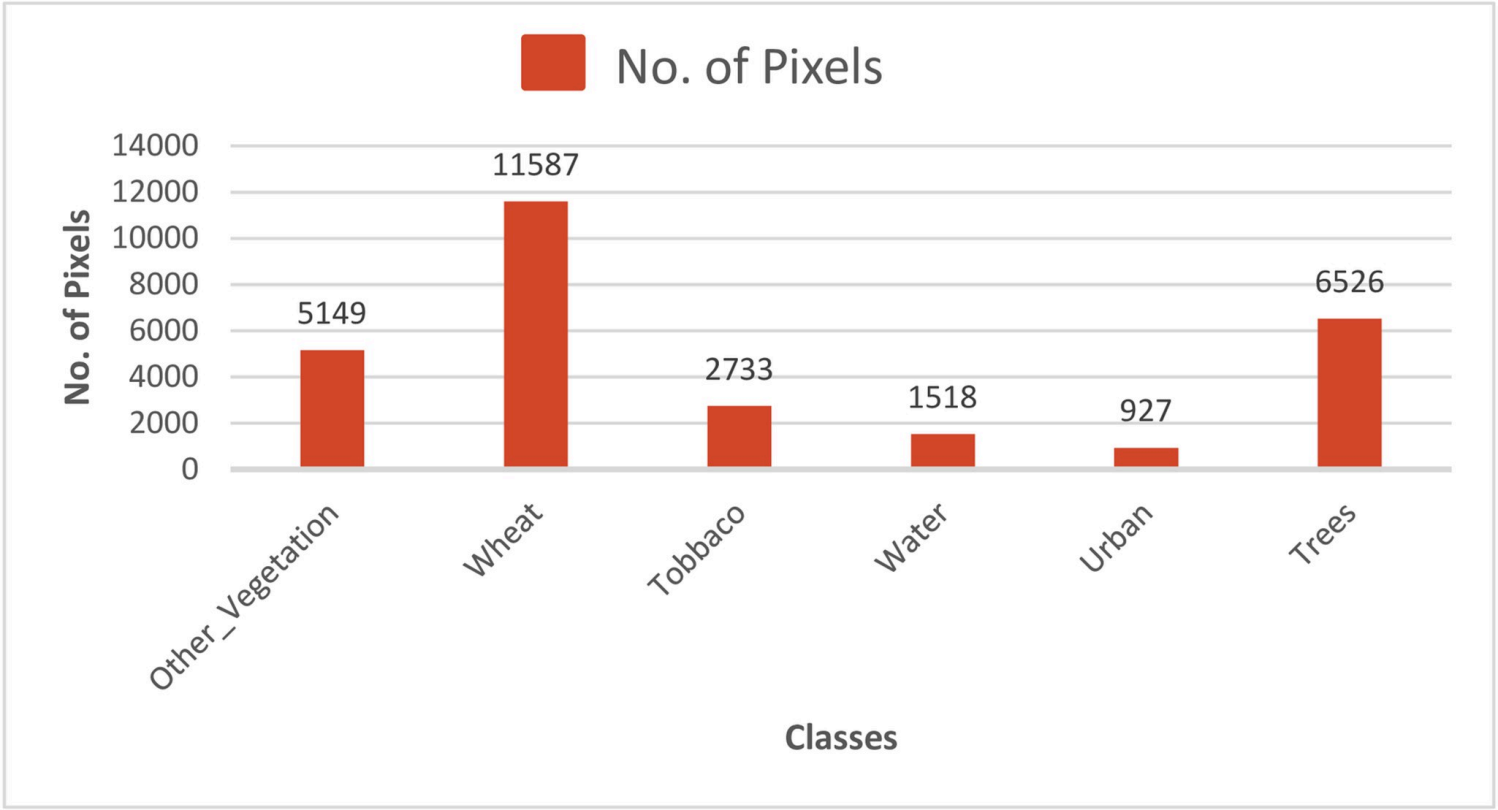
Number of pixels collected for each class.

The samples obtained during the pilot region survey were divided into two subsets, namely training and testing sets. The datasets for training and research have an average representation of 80 percent and 20 percent of the study dataset. Furthermore, a 15 percent training set data is segregated as a validation set. In addition, the stratified k-fold technique is used with 5 folds for the statistical validation and performance analysis of our proposed model. The dataset is composed of multi-spectral imagery from 2 separate Sentinel-2 and Planet-Scope satellites. Sentinel-2 offers 13 different spectral bands with a 5-day temporal resolution, while Planet-Scope provides four different 1-day temporal resolution spectral bands. Spectral bands were chosen on the basis of their capacity and the degree to which the relative content of information was provided in relation to our target research work. For Planet-Scope and Sentinel-2, three bands of Red, Green and Near-Infrared having 3 m and 10 m spatial resolution respectively, were chosen. The blue band is discarded because it is not helpful in crop classification and is very susceptible to atmospheric particles, such as dust and clouds [34]. For each ground class, the number of pixels collected through ground survey is presented in Fig 5.

### Data preprocessing

The remotely sensed imagery acquired (Fig 4), was spatially re-sampled using bi-linear interpolation due to difference in band resolutions of both Sentinel-2 and Planet-Scope. All Sentinel-2 bands were resampled to Planet-Scope resolution of 3 metres. For both Sentinel-2 and Planet-Scope satellite imagery, the Normalized Difference Vegetation Index (NDVI) spectral index was measured separately. The NDVI is helpful in green vegetation detection and is widely used in literature as input. The measured NDVI index is packed with acquired multi-spectral imagery as an extra layer. Further, both the Sentinel-2 and Planet-Scope images at different timestamps of the area of interest are temporally stacked, and is the final image used by our proposed model. More precisely, the resulting stacked temporal imagery consists of 20 total bands, 4 of which are Planet-Scope bands, and 16 of which are Sentinel-2 satellite imagery.

The spectral intensities of bands are provided in 12 bit, with values ranging from 0-4096, whereas the spectral indices such as NDVI in the range -1 to +1. The values are preprocessed using robust scaling of all the spectral intensities in the same range. For neural networks, the data in the same range optimises the training process, and the loss and accuracy merges quickly. It also curbs any bias produced by the different scales of the input features. The imbalance of the dataset as seen in Fig 5, is dealt with the weighted loss. Weighted loss penalizes the misclassification made by the minority class by setting higher class weight and at the same time reducing weight for the majority class. Further, in order to reduce the influence of the outliers (erroneous data), robust scaler based on percentiles is used. The robust scaler is therefore not influenced by a fewer number of very large marginal outliers.

## Deep learning model architecture

**Fig 6:**
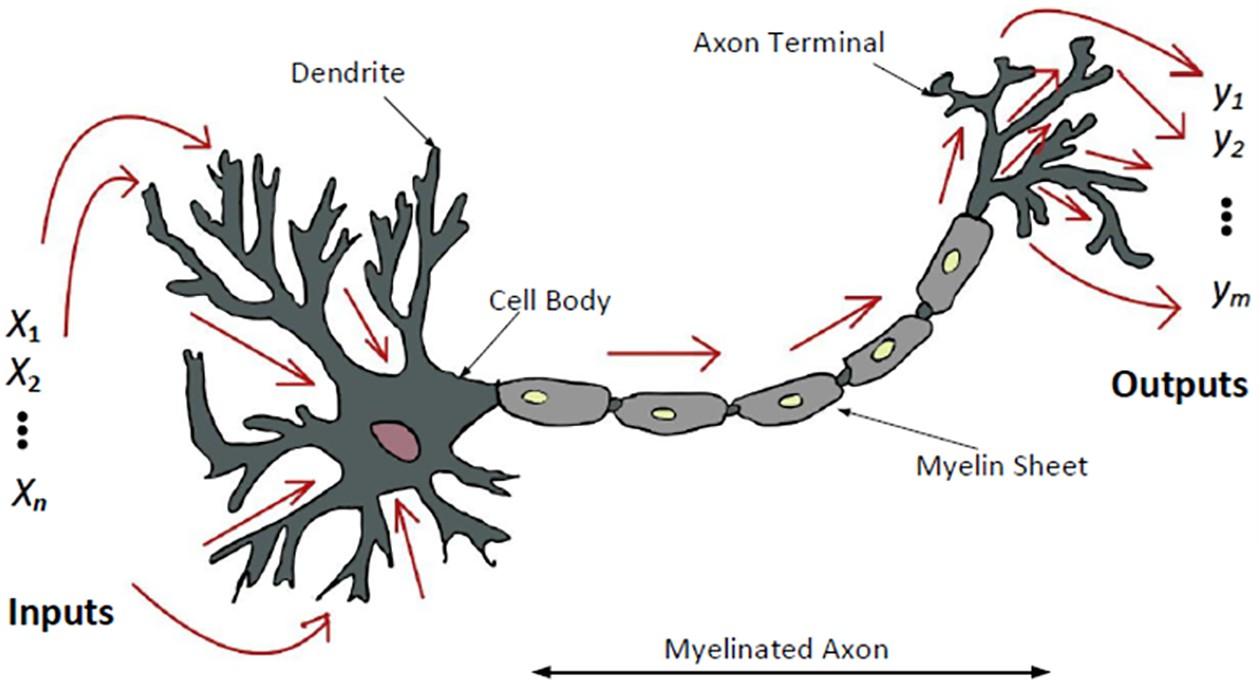
A biological neuron: It accepts multiple inputs *x* from other perceptrons, and produces a positive output *y* if the total total evidence is higher the threshold of neurons.

ANNs originated from a simple multi-layered network of perceptrons, where each perceptron is modeled against a biological neuron [35]. A biological neuron as depicted in Fig 6, receives inputs from other neurons using dendrites and outputs signals using axons. If the inputs signals have higher intensity than the threshold, it fires up or activates, resulting in a positive charge at the output terminals. Similarly, artificial neurons or perceptrons accepts input *x* and produces an output *y*. Each input is multiplied by a weight *w* and a bias *b* is added to it. The linear combination of weights, input and bias is passed through a non linear activation function such as sigmoid or relu, for producing the output.

$$\begin{array}{c} Z=W_{1}X_{1}+W_{2}X_{2}+\ldots +W_{n}X_{n}+b \end{array}$$ (1)

$$\begin{array}{c} Y^{{}^{\prime }}=\text{Sigmoid}(Z) \end{array}$$ (2)

$$\begin{array}{c} H_{p}\left(q\right)=-\frac{1}{N}\sum_{i=1}^{N}y_{i}\cdot \;\text{log}\;(p(y_{i}))+(1-y_{i})\cdot \;\text{log}\;(1-p(y_{i})) \end{array}$$ (3)

Where as *X*_1_, *X*_2_, *X*_3_….*X*_*n*_ are inputs and *Y*_1_, *Y*_2_, *Y*_3_….*Y*_*n*_ are the outputs.

ANNs were developed to mimic the human intelligence and are becoming popular in machine learning algorithms. With the advancement of technology, however alot of data was produced for which ANN has fewer limitations, like greater input parameters (computationally unfeasible), exploding and vanishing gradient [36] of the back-propagation algorithms and the inability of ANN to process sequential data. The solution to these limitations results in Convolutional Neural Network (CNN) and Recurrent Neural Network (RNN) [37]. The CNN algorithms were the best at processing large amount of data and dealing with higher input parameters for input and kernel convolutions. The RNNs were specifically developed to solve the inability of the ANN model in handling sequential data such as speech, text and videos. However, as the number of RNN layers increases, it was observed that RNN falls into the trap of vanishing gradient problem. The solution to this led the researchers to LSTM [38], a different variant of RNN with memory.

### Long short term memory network

**Fig 7:**
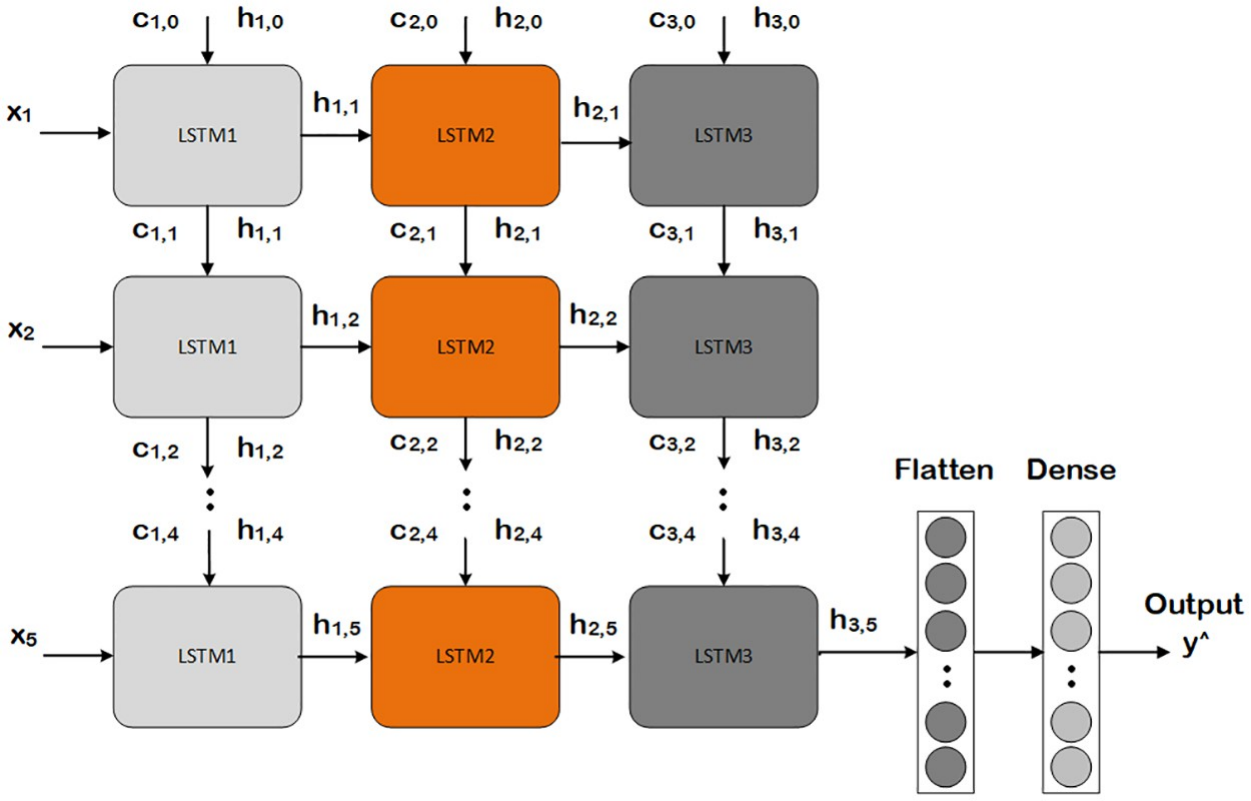
Proposed LSTM model.

LSTM architecture is derived from the RNN architecture, and it is successfully utilized in temporal data tasks [39]. The consistent high performance of LSTM network is due to its ability of capturing long term dependencies in the data. The architecture structure used in our experimentation work is presented in Fig 7. The model consists of a memory cell that holds a current state over different sequential instances and non-linear dependencies, controlling entry and exit of information to the cell. The *X*_*t*_ is the input vector to our model architecture depicts the input at time *t*, *C*_*t*_ is the memory of the current block, and *h*_*t*_ is the output of the current block, while *C*_*t*−1_ and *h*_*t*−1_ are the memory and output from previous block. The vector operations that are performed in LSTM network is element-wise concatenation (+) and element-wise multiplication (*). For calculating the associated non-linearities, hyperbolic tangent (tanh) and Sigmoid (σ) activation functions are used.

### Convolutional long-short term memory

ConvLSTM is a variant of the LSTM network and captures spatial features in multi-dimensional data by convolution process. As LSTM input data is one-dimensional, it is not suitable for spatial sequence data such as video, satellite, and radar image dataset. ConvLSTM is designed for 3-D input data. Shi *et al*. developed ConvLSTM primarily for the problem of spatiotemporal sequence forecasting [40]. At each gate in the LSTM cell, ConvLSTM replaces matrix multiplication with convolution operation (ConvLSTM has convolutional structures in both the input-to-state and state-to-state transitions). The input dimensions of the data is maintained in ConvLSTM cell, and is not being just a function of 1-D vector with features. ConvLSTM has convolution embedded in the architecture of LSTM network. In ConvLSTM architecture, the model passes previous hidden state to the next step of sequence. Therefore, holding information on previous data which the network has already seen before is used for making decisions. In other words, the data order is extremely important.

**Fig 8:**
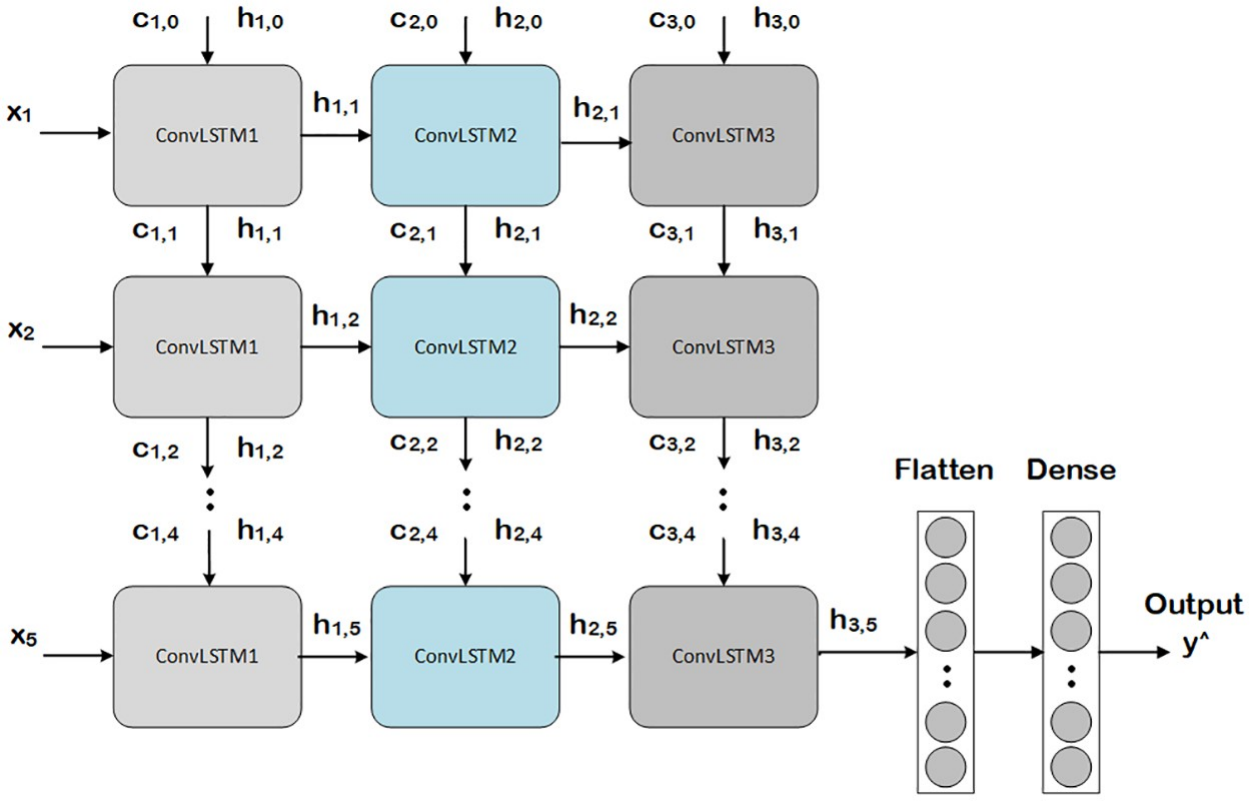
Proposed ConvLSTM model.

In our case, the data is spectro-temporal and we proposed a model based on ConvLSTM layers as presented in Fig 8.

The associated equations of ConvLSTM are given below, where * denotes the convolution operator and the element-wise product:

$$\begin{array}{c} \begin{array}{c} i_{t}=\sigma (W_{xi}*X_{t}+W_{hi}*H_{t-1}+W_{ci}⊙C_{t-1}+b_{i})\\ f_{t}=\sigma (W_{xf}*X_{t}+W_{hf}*H_{t-1}+W_{cf}⊙C_{t-1}+b_{f})\\ C_{t}=f_{t}⊙C_{t-1}+i_{t}⊙\text{tanh}(W_{xc}*X_{t}+W_{hc}*H_{t-1}+b_{c})\\ o_{t}=\sigma (W_{xo}*X_{t}+W_{ho}*H_{t-1}+W_{co}⊙C_{t}+b_{o})\\ H_{t}=o_{t}⊙\text{tanh}(C_{t}) \end{array} \end{array}$$ (4)

The experimental results of our method are presented in Table 1. The model is trained for different layers, filters and various batch sizes. The training and validation set accuracies are evaluated for the different given parameters in Table 1.

**Table 1:** Experimentation results of ConvLSTM with 32-Filters.

Filter Size 32-Filters								
Layer 1			Layer 2			Layer 3		
Batch size	TA	VA	Batch size	TA	VA	Batch size	TA	VA
16	0.95709	0.96754	16	0.94954	0.95805	16	0.95397	0.96497
32	0.95474	0.95481	32	0.94833	0.95453	32	0.97661	0.97700
64	0.94308	0.95228	64	0.91698	0.92718	64	0.93912	0.94831
128	0.91631	0.92405	128	0.89542	0.90277	128	0.89174	0.90172
256	0.89076	0.89507	256	0.86204	0.86367	256	0.86661	0.86673

Our proposed model architecture has three ConvLSTM layers. The inputs (*x*1, *x*2, *x*3, *x*4, *x*5) are fed to the model at time (*t*1, *t*2, *t*3, *t*4, *t*5) respectively. The outputs to each of the current input *x* and previous output is the recurrent input (h) for the next layer. The final output of the third layer is flattened and is fed to a dense neural network yielding the class probabilities for all the inputs at all temporal instances. The input of a ConvLSTM is a set of images over time as a 5D tensor with shape (samples, time steps, channels, rows, cols).

## Results and discussion

**Fig 9:**
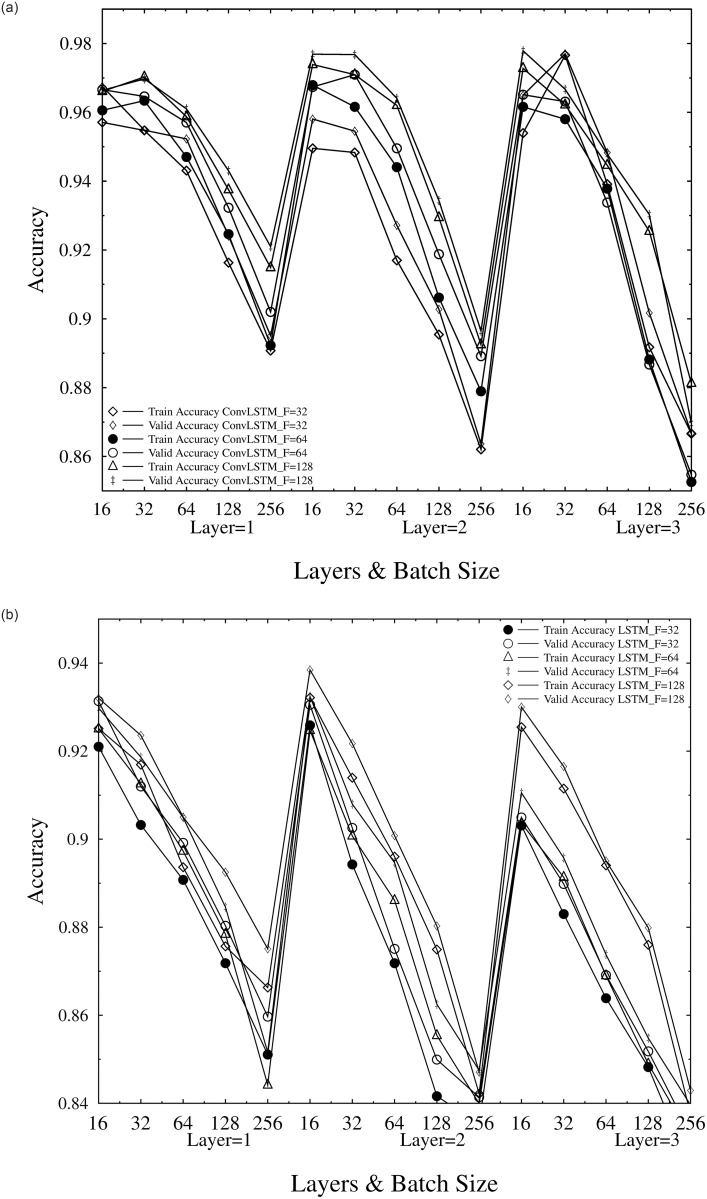
(a). Experimentation results plot of ConvLSTM (b). Experimentation results plot of LSTM.

Experimentation for ConvLSTM over various parameters has been performed and the results as training (TA) and validation accuracy (VA) are shown in Fig 9(a) and 9(b). The graphs represents the analysis of ConvLSTM and LSTM for comparison purposes using different hyper-parameters (Batch size, Number of layers and Number of filters). It can be clearly seen that ConvLSTM outperforms LSTM by the training and validation accuracy of 97.278% and 97.788% respectively, using 128 Filters, 3 Layers and 16 Batch size. With the maximum accuracy achieved by LSTM using a batch size of 16, 2 layers and 128 filters is recorded to be 93.215% for TA and 93.845% for VA. A total of 45 experiments were conducted using varying hyper-parameters. Results in Training Accuracy (TA) and Validation Accuracy (VA). Tables 1–3 lists the results received using ConvLSTM. while the outcomes of LSTM are tabulated in Tables 4–6.

**Table 2:** Experimentation results of ConvLSTM with 64-Filters.

Filter Size 64-Filters								
Layer 1			Layer 2			Layer 3		
Batch size	TA	VA	Batch size	TA	VA	Batch size	TA	VA
16	0.96058	0.96659	16	0.96790	0.96736	16	0.96160	0.96511
32	0.96339	0.96462	32	0.96160	0.97095	32	0.95801	0.96311
64	0.94703	0.95703	64	0.94406	0.94954	64	0.93782	0.93379
128	0.92460	0.93227	128	0.90616	0.91877	128	0.88816	0.88670
256	0.89228	0.90200	256	0.87893	0.88913	256	0.85257	0.85471

**Table 3:** Experimentation results of ConvLSTM with 128-Filters.

Filter Size 128-Filters								
Layer 1			Layer 2			Layer 3		
Batch size	TA	VA	Batch size	TA	VA	Batch size	TA	VA
16	0.96608	0.96638	16	0.97389	0.97689	16	0.97278	0.97788
32	0.97027	0.96972	32	0.97095	0.97679	32	0.96218	0.96670
64	0.95874	0.96100	64	0.96202	0.96406	64	0.94461	0.94789
128	0.93751	0.94303	128	0.92942	0.93435	128	0.92544	0.93030
256	0.91488	0.92095	256	0.89249	0.89644	256	0.88128	0.86958

**Table 4:** Experimentation results of LSTM with 32-Filters.

Filter Size 32-Filters								
Layer 1			Layer 2			Layer 3		
Batch size	TA	VA	Batch size	TA	VA	Batch size	TA	VA
16	0.92101	0.93134	16	0.92586	0.93051	16	0.90309	0.90493
32	0.90323	0.91196	32	0.89425	0.90256	32	0.86298	0.87181
64	0.89075	0.89916	64	0.87181	0.87507	64	0.85386	0.85713
128	0.87182	0.88032	128	0.8416	0.84992	128	0.84821	0.8518
256	0.85106	0.85968	256	0.83533	0.84138	256	0.825215	0.83294

**Table 5:** Experimentation results of LSTM with 64-Filters.

Filter Size 64-Filters								
Layer 1			Layer 2			Layer 3		
Batch size	TA	VA	Batch size	TA	VA	Batch size	TA	VA
16	0.92507	0.92979	16	0.92471	0.93242	16	0.90359	0.91053
32	0.91258	0.91861	32	0.90073	0.90797	32	0.89135	0.89574
64	0.89728	0.9049	64	0.8861	0.89464	64	0.8689	0.87378
128	0.8784	0.88456	128	0.8554	0.86251	128	0.84895	0.85481
256	0.84415	0.85161	256	0.83991	0.8475	256	0.83226	0.83959

**Table 6:** Experimentation results of LSTM with 128-Filters.

Filter Size 128-Filters								
Layer 1			Layer 2			Layer 3		
Batch size	TA	VA	Batch size	TA	VA	Batch size	TA	VA
16	0.92513	0.93202	16	0.93215	0.93845	16	0.92545	0.93001
32	0.92692	0.93357	32	0.91395	0.92172	32	0.91149	0.9165
64	0.89362	0.90502	64	0.89602	0.90078	64	0.89403	0.89506
128	0.90565	0.91249	128	0.87493	0.88028	128	0.87599	0.87987
256	0.86629	0.87508	256	0.84216	0.84706	256	0.83746	0.8429

The reason for choosing these parameters is because, as the number of layers increases, the depth of the model also increases, which impacts the computational cost. Hence this is an important parameter to be studied for its performance impact on the model. Furthermore, batch sizes are chosen as per the norms of the deep learning research community which are usually selected as 16, 32, 64, 128 and 256. Moreover, the filter extracts the features from the data. As the number of filters increases more features from the data are extracted which are the model used for learning and prediction. Hence, it is also important to study the impact of this parameter on the model performance. So the chosen experimental setup of these parameters is for evaluating the impact of these various parameters to find out the best setting in terms of model performance.

### ConvLSTM

In our implemented models, in order to study the effect of layers, we have used three ConvLSTM layers.

Although the variation between TA and VA (which shows the model over-fitting) are not smooth. However, the overall difference decreases as the number of layers increases, showing the performance improvement of the model.

For analyzing the effect of number of filters, we have used three setting of number of filters for 32-filter, 64-filters and 128-filters in our experimental setup. It is analysed that for 128-filters, the training accuracy is slightly greater than 64-filter and 32-filter, but the validation accuracy of 64-filters is greater then 32 and 128-filters, over-fitting the learning process. The performance of the model is also compared over varying batch sizes, and it was found that the performance of model over the training accuracies increases then decreases. The following assumptions can be elaborated from the results of ConvLSTM.

- TA and VA of 97.027% and 96.972% is achieved using Batch size of 32 with 1 layers and 128 filters.
- An accuracy of 97.389% for training and 97.689% for validation is recorded for Batch size 16, 2 layers and 128 filters.
- TA and VA of 97.095% and 97.679% is achieved using Batch size of 32, with 2 layers and 128 filters.
- 97.278% and 97.788% of TA and VA has been observed by experimenting on Batch size of 16, 3 layers and 128 filters.
- The maximum TA and VA accuracy recorded using batch size of 32, 3 layers and 32 filters is 97.661% and 97.700% respectively.

The maximum training accuracy is on batch size is 32, which validates the traditional setting of batch size to 32. However for VA, the shift is not smooth but fluctuates apart from layer 3 and 128-filters.

## LSTM

The same hyper-parameters were used for experimentation using LSTM. With the TA and VA achieved by LSTM is 93.215% and 93.845% respectively, hence the model under performs as compared to ConvLSTM. It can also be seen from Fig 9(b) that the model shows mediocre performance for 32 Batch size through out the experiments.

## Validation criteria

Relying on overall accuracy is never ideal for performance evaluation of a classifier. In our experiments the following parameters were considered for classification evaluation.

### Precision

Precision explains the fidelity of the classifier, as it is calculated by taking ratio between true positive to the sum of the true positive and false positive. Under the classification of land cover and land use (LCLU) precision is best known as user accuracy where;

$$Precision=\frac{\text{True}\;\text{Positive}}{\text{True}\;\text{Positive}\;\text{+}\;\text{False}\;\text{Positive}\;}$$

### Recall

Recall provides information on the classifier’s perfection, defined as the ratio of true positives to the sum of true positives and false negatives for each class. Therefore LCLU recall is simply known as producers accuracy where;

$$Recall=\frac{\text{True}\;\text{Positive}}{\text{True}\;\text{Positive}\;\text{+}\;\text{False}\;\text{Negitive}\;}$$

### F1-score

It is the weight harmonic mean of precision and recall ranging from 1.0 to 0.0 where 1.0 is a good F1 score and 0.0 is worst case.

$$F1Score=2*\frac{\left(\text{Recall}\;*\;\text{Precision}\right)}{\left(\text{Recall}\;\text{+}\;\text{Precision}\;\right)}$$

### Overall-accuracy

It is the ratio of sum of all correctly classified training data pixels to the total number of training data pixels.

$$OverallAccuracy=\frac{\left(\text{Number}\;\text{of}\;\text{all}\;\text{correctly}\;\text{classified}\;\text{pixel}\right)}{\left(\text{Total}\;\text{number}\;\text{of}\;\text{Pixels}\;\right)}*100$$

The selected best model through experimentation is applied on test data. Table 7 shows the confusion matrix of the model classification of the test data. Table 8 presents the classification report, explaining the model performance over test data. The performance of the model is measured through precision, recall and F1-score as well the overall accuracy of the model over test data. As can be seen from Table 7, the performance of model is high in correct identification of the actual data, except for Other vegetation. where the precision is 0.67. This is mainly due to the fact that Other Vegetation class includes a mix of different vegetation, and for the model it is natural to get confused among different classes. Hence, others class pixels such as Tobacco, Wheat and Trees are having high spectral similarity with Other Vegetation class are classified in Other Vegetation. The Precision metric is the highest for Trees class, which is due to its spectral dissimilarity from the lively fresh green vegetation. The Recall metric tell us about the correct identification of reference data. The minimum Recall value is 0.80 for Wheat class due to its similarity with the Tobacco and Other Vegetation classes and is evident from the confusion matrix. Further, in order to check the precision and robustness of our model in biased scenario (as our dataset), we have calculated F1-Score. F1-score is the harmonic mean of Precision and Recall with a range of [0, 1], and is considered more better metric for an unbalanced dataset. The F1-Score is minimum for Other Vegetation class, and is maximum for the Trees class. Overall classification accuracy for our model is 0.913.

**Table 7:** Confusion matrix of ConvLSTM.

Confusion Matrix: Predicted Class						
	Other Veg 0	Wheat 1	Tobacco 2	Water 3	4 Urban	Trees 6
Other Veg 0	4946	130	0	2	0	71
Wheat 1	117	11348	0	1	0	121
Tobacco 2	0	0	2733	0	0	0
Water 3	0	0	0	1515	0	3
Urban 4	0	0	0	0	927	0
Trees 6	162	69	0	0	0	6295

**Table 8:** Classification report explaining performance of ConvLSTM over test data.

Classification Report:				
	Precision	Recall	F1-Score	Support
Other Veg 0	0.95	0.96	0.95	5149
Wheat 1	0.98	0.98	0.98	11587
Tobacco 2	1.00	1.00	1.00	2733
Water 3	1.00	1.00	1.00	1518
Urban 4	1.00	1.00	1.00	927
Trees 6	0.97	0.96	0.97	6526
**Accuracy**	0.98			28440
**Macro Average**	0.98	0.98	0.98	28440
**Weighted Average**	0.98	0.98	0.98	28440
**Classification Accuracy**:	0.9758			

**Fig 10:**
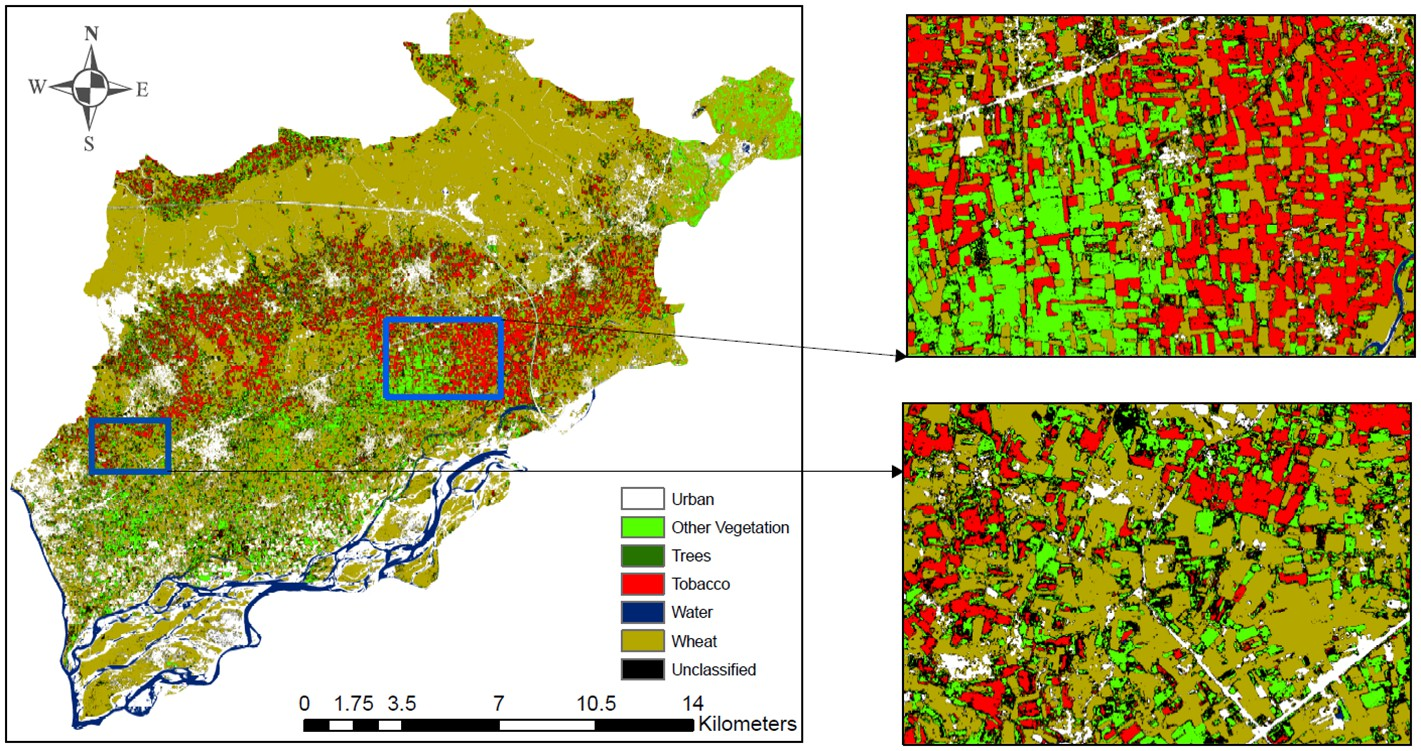
ConvLSTM classified map (generated through our own model and no copyright permission is required) of the region of interest.

The land-use land-cover classification map are shown in Fig 10.

## Conclusion

By analyzing the results and affects of various parameters over the performance of ConvLSTM, we concluded that as the batch size increases within a layer, the performance of ConvLSTM model improves in terms of generalization and speed, although maximum training accuracy is achieved when the batch size is 32. On the other side, as the number of layers increases from one to two, there is a slight increase in the training accuracy of the model. However, as the layers increases from two to three, there is no significant change in training accuracy, however validation accuracy varies. The increase in number of filters negatively impacts the generalization of classifier. Although our model has a high training accuracy over 64-filter, and for the overall layers, however the validation accuracy is always low, other than for the parameters with 64 and 128 number of filters.
